# Implant rehabilitation of a posterior maxilla with 4‐mm long implants splinted to a 10‐mm long implant in a patient with osteopenia taking antiresorptive drugs: A 5‐year follow‐up case report

**DOI:** 10.1002/ccr3.7291

**Published:** 2023-05-20

**Authors:** Katja Povšič, Čedomir Oblak, Michel Dard, Rok Gašperšič

**Affiliations:** ^1^ Department of Oral Medicine and Periodontology, Faculty of Medicine University of Ljubljana Ljubljana Slovenia; ^2^ Department of Prosthodontics, Faculty of Medicine University of Ljubljana Ljubljana Slovenia; ^3^ College of Dental Medicine Columbia University New York USA; ^4^ Institut Straumann AG Basel Switzerland

**Keywords:** alveolar bone loss, dental implants, maxilla, osteopenia, short dental implants, survival rate

## Abstract

The report describes the rehabilitation of a maxillary arch with limited bone volume in a 67‐year‐old female taking antiresorptives due to osteopenia. One 10‐mm and two extra‐short 4‐mm implants were inserted, and implant‐supported splinted crowns were fabricated. The 5‐year follow‐up showed stable bone levels, despite poor initial stability (ISQ: 14–51).

## 1 INTRODUCTION

Low bone density and deficient bone dimensions present a challenge for implant insertion in the posterior maxilla. In cases where the volume of residual bone is reduced to less than 8 mm due to sinus pneumatisation and/or extensive postextraction bone resorption, several surgical options may be considered during the formulation of a treatment plan for implant‐supported prosthetic rehabilitation: a transcrestal (closed) sinus lift combined with the use of standard‐length implants; a simultaneous or delayed lateral (open) sinus lift; or the use of short (<8 mm) and ultrashort (<6 mm) implants.^1^

Short and extra‐short implants have slowly become recognized by patients and clinicians in the past years. They require less aggressive surgical techniques and result in shorter intraoperative periods, reduced morbidity and lower treatment prices.^2^ The predictability of mid‐term outcomes in single‐tooth and splinted short‐implant‐supported rehabilitations can be attributed to recent technological improvements and adjusted treatment protocols. Besides the lower incidence of comorbidities and complications in comparison to sinus lift procedures,^3^ the rationale for using short implants is based on the observation that the maximum stress concentration occurs on the first 5–6 mm of the implant, irrespective of its length.^4^ After loading, short implants may experience more failures than standard‐length implants because of their biomechanical shortcomings, particularly in the maxilla.^5^

In general, osteoporosis or low‐dose oral antiresorptive drug (ARD) intake for its treatment does not compromise implant therapy; that is, patients taking oral ARDs do not lose more implants, experience inferior osseointegration or get more implant‐related complications/failures compared to patients without a history of such medication.^6^, ^7^, ^8^ Nevertheless, if implant loss occurs, it is more likely to occur within a short time after loading (early loss^9^) and in the posterior maxilla.^6^, ^8^ In addition, posterior dental implants are difficult to access and clean, making the development of peri‐implantitis more likely.^10^ No information, however, exists regarding peri‐implant marginal bone levels in patients taking ARDs.^6^ To the best of our knowledge, success and failure rates of short implants in patients with osteoporosis or patients taking ARDs have not yet been evaluated.

In our recent case series study,^11^, ^12^ we tested the possibility of rehabilitating posterior edentulous maxillary areas with 4‐mm extra‐short implants splinted to 10‐mm implants. We have already presented the treatment protocol^11^ as well as the 1‐^12^ and 3‐year^13^ results. The survival and success rates were found to be 100% in all 11 cases. The present case report describes the successful rehabilitation and 5‐year follow‐up period of a patient with poor maxillary bone quality, extremely low primary implant stability, osteopenia and a 9‐year history of preventive ARD therapy.

## 2 MATERIALS AND METHODS

This paper describes a case of a 67‐year‐old woman with a medical record of primary osteopenia. Her medical record revealed primary osteopenia diagnosed by dual x‐ray absorptiometry (DXA) with a T‐score of −1.9.^14^ She had been undergoing preventive ARD treatment for the past 9 years: the first 7 years with alendronate, 70 mg p.o./week (Fosavance®, Merck Sharp & Dohme BV) and the last 2 years with denosumab 30 mg s.c./6 months (Prolia®, Amgen). She was also taking vitamin D and calcium supplements. Implant insertion was performed 5 months after the last denosumab injection and 6 months after extraction of teeth #2 and #5.

**FIGURE 1:**
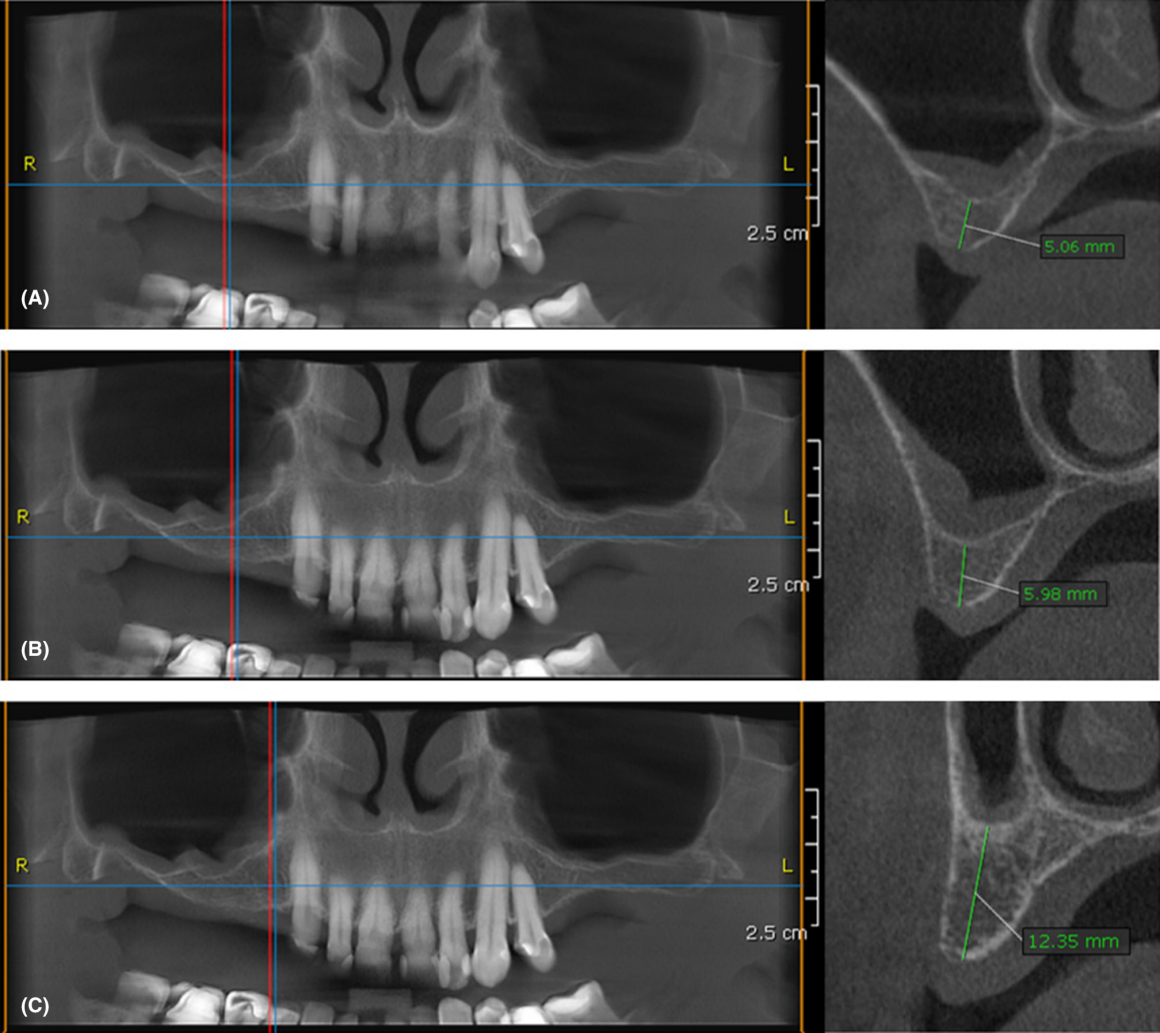
CBCT scan of the maxilla before implant placement. (A) Bone dimensions at the edentulous site of tooth no. 16. (B) Bone dimensions at the edentulous site of tooth no. 15. (C) Bone dimensions at the edentulous site of tooth no. 14.

The clinical evaluation revealed a bilaterally shortened maxillary dental arch. Her natural dentition was preserved from tooth #6 to tooth #12. The vertical alveolar ridge dimension seemed satisfactory, yet the CBCT scan revealed an expanded maxillary sinus. The reduced vertical bone dimension measured 5.06 and 5.98 mm at the edentulous sites of former teeth no. #3 and #4, respectively. The vertical bone dimension at the edentulous site of former tooth #5 measured 12.35 mm (Figure 1). The alveolar ridge width in the right edentulous maxillary region measured 7 mm or more. In the mandible, the dental formula was from tooth #19 to tooth #31. Tooth #20 had been replaced with a titanium (Ti) implant 12 years ago and tooth #30 had been restored with a porcelain‐fused‐to‐metal crown. The patient's oral hygiene was good (full mouth plaque score: 18%), gingival inflammation was negligible and periodontitis‐associated bone loss was minimal. There was no sign of mucosal pathology.

Approval for the planned treatment as part of the case series study was obtained from the Republic of Slovenia's National Ethics Committee (No. 30/10/2015). Written informed consent was obtained from the patient prior to treatment and paper publication. The treatment plan was to provide her with 2 extra‐short 4‐mm implants in the edentulous sites of former teeth #3 and #4 and one 10‐mm implant at the edentulous site of former tooth #5. Restoration with splinted crowns supported by these three implants was planned to occur 6 months after implant insertion. An acrylic surgical guide for a pilot drill was fabricated to obtain optimal position and inclination of the implants. After the application of local anesthesia (Ultracain®, Hoechst), a midcrestal incision was made and a full‐thickness flap reflected. The standard implant bed preparation sequence was followed as recommended by the manufacturer (Institut Straumann AG). The profile drill was omitted for the 4‐mm implant beds. Finally, one 10‐mm × 4.1‐mm diameter and two 4‐mm × 4.1‐mm diameter (0.8 mm thread pitch and a 1.8 mm polished collar) tissue‐level (Standard Plus, titanium‐zirconium [Ti‐Zr] alloy, SLActive®) implants (Institut Straumann AG) were manually inserted. Implant stability was measured immediately after insertion using a hand ranchette and Ostell® equipment (Integration Diagnostics). Finally, closure screws (height: 2 mm) were inserted onto the implants and flaps were approximated and sutured using 5–0 non‐resorbable polypropylene sutures (Prolen®). In the same session, one 10‐mm × 4.1 mm bone‐level (BL) implant was inserted at the edentulous site of former tooth #13. A soft diet and chlorhexidine rinse 2×/day was recommended for 2 weeks. Diclofenac 75 mg p.o./8 h (Naklofen duo®, Krka) was prescribed for 2 days. Secondary herpetic lesions appeared in the operative region 4 days after implant insertion; valacyclovir 500 mg p.o./12 h (Valtrex®, GSK) was prescribed for 14 days. The sutures were removed 2 weeks after implant insertion and the implant position checked by radiograph.

After 6 months, the implants were deemed ready for prosthetic rehabilitation. Following an X‐ray of the three implants, their stability was tested using resonance frequency analysis (Osstell®, Integration Diagnostics). Then, an implant‐level definitive open tray impression was made with polyether impression material (Impregum®, 3M ESPE). The metal framework was designed and manufactured by a computer‐aided software using Coron®, a cobalt‐chromium alloy (Institut Straumann AG). Finally, the patient was restored with a metal‐ceramic fixed dental prosthesis cemented onto individually tailored original abutments, which were screwed to the three implants with a torque of 35 Ncm.

**FIGURE 2:**
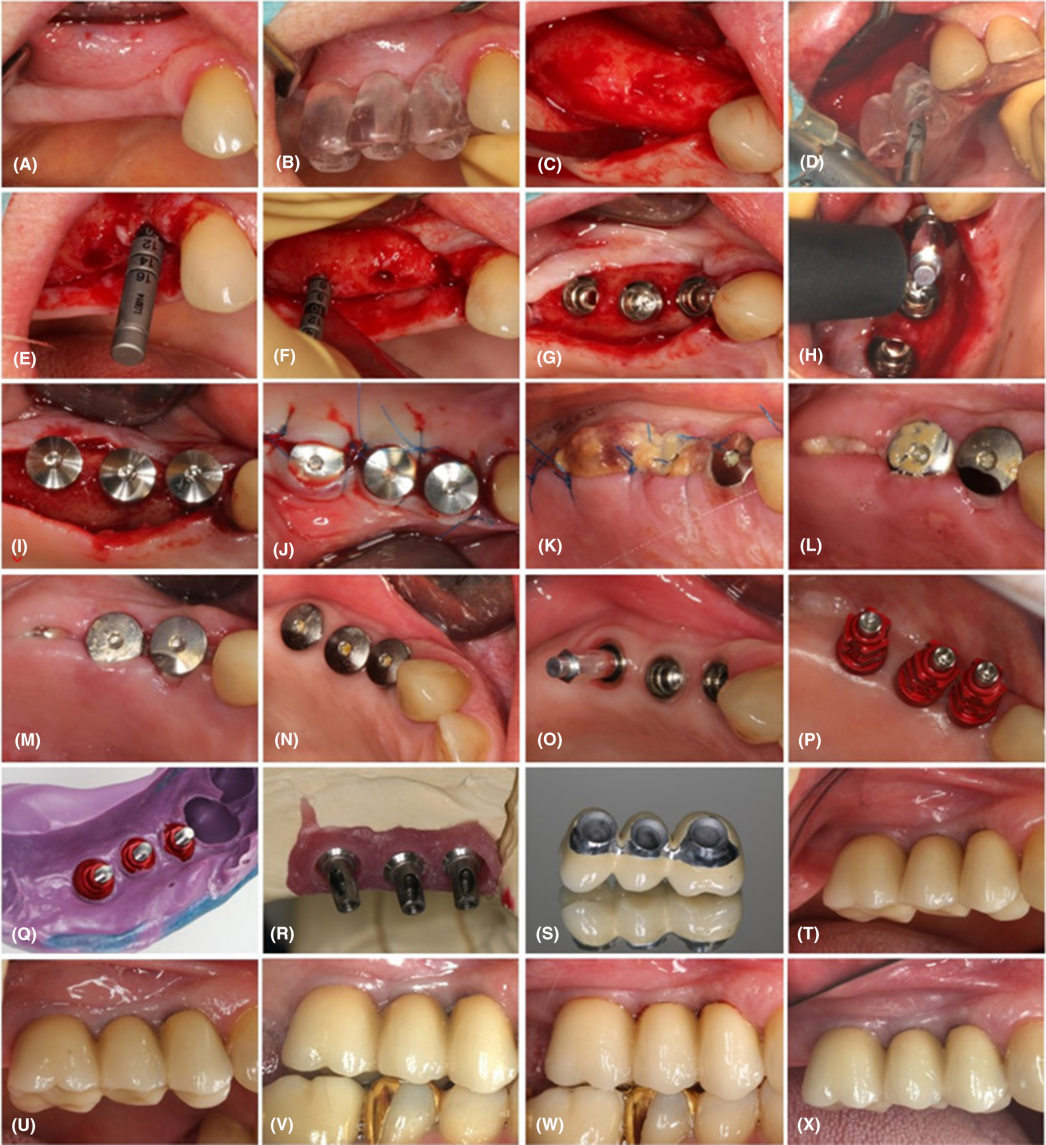
Workflow of prosthetic rehabilitation. (A) Maxillary arch at baseline. (B) Surgical guide for dental implant placement. (C) Reflection of full‐thickness flap at the site if implant insertion. (D) Preparation of the implant beds using a surgical guide. (E) Validation of implant bed adequacy at edentulous site of former tooth no. 14. (F) Validation of implant bed adequacy at edentulous site of former tooth no. 16. (G) Insertion of implants at edentulous sites of former teeth no. 14, 15, and 16. (H) Measurement of implant stability immediately after insertion using Ostell® equipment (Integration Diagnostics). (I) Insertion of closure screws onto implants. (J) Suturing of the full‐thickness flaps. (K) Surgical site 4 days after implant insertion; secondary herpetic lesions on the palate. (L) Surgical site following suture removal, 14 days after implant insertion. (M) Surgical site 1 month after implant insertion. (N) Surgical site 6 months after implant insertion. (O) Measurement of implant stability 6 months after implant insertion using Ostell® equipment (Integration Diagnostics). (P) Positioning of open‐tray copings for the registration procedure. (Q) Implant position registration utilizing open‐tray copings and polyether impression material (Impregum®, 3 M ESPE). (R) Transfer of implant position onto a plaster cast. (S) The metal framework made of Coron®, a cobalt‐chromium alloy (Institut Straumann AG). (T) Prosthetic restoration after cementation. (U) Prosthetic restoration 1 year after cementation. (V) Prosthetic restoration 2 years after cementation. (W) Prosthetic restoration 3 years after cementation. (X) Prosthetic restoration 4 years after cementation.

The patient was thereafter recalled every 4–6 months. At each visit, we reinforced her oral hygiene instructions, and meticulously cleaned any tooth deposits. Every 12 months, a detailed periodontal examination (evaluation of full mouth of plaque score, gingival bleeding index, pocket probing depth, recession, bleeding on probing) was performed in addition to a radiographic examination (without bite blocks or standardization methods) of the implants, whereby radiographic bone levels were accurately measured. The clinical follow‐up is presented in Figure 2.

## 3 RESULTS

Based on its radiographic appearance, bone quality was estimated as type III at the edentulous sites of former teeth #4 and #5 and type IV at the edentulous site of former tooth #3. Insertion torques of all implants were below 15 Ncm, and initial ISQ values measured 14, 49, and 52 for implants at edentulous sites #3, #4, and #5, respectively.

Four days following implant insertion, the patient complained of pain at the right maxillary surgical site. A detailed clinical examination revealed ruptured vesicles and fibrine exudate, characteristic for secondary herpetic mucosal lesions. Fourteen days later, the lesions were successfully cured, and sutures removed. After 6 months, the ISQ values for implants at edentulous sites #3, #4, and #5 increased to 56, 51, and 60, respectively. Slight marginal bone resorption was noticed, typical for tissue level implants.

**FIGURE 3:**
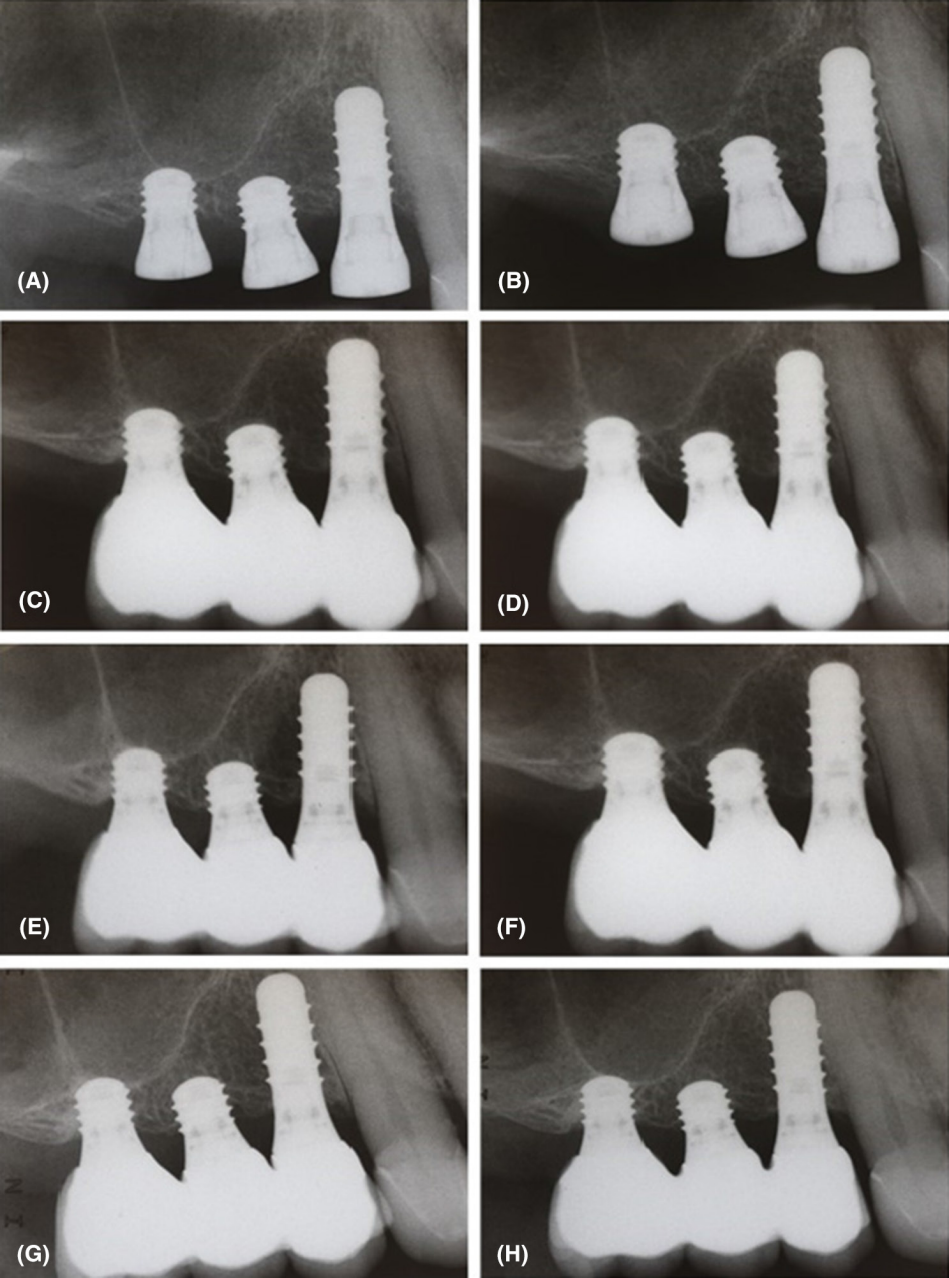
Radiographic examination of implants: (A) immediately after insertion, (B) 6 months after insertion, (C) immediately after crown cementation, (D) at the 1‐year follow‐up visit, (E) at the 2‐year follow‐up visit, (F) at the 3‐year follow‐up visit, (G) at the 4‐year follow‐up visit, (H) at the 5‐year follow‐up.

**FIGURE 4:**
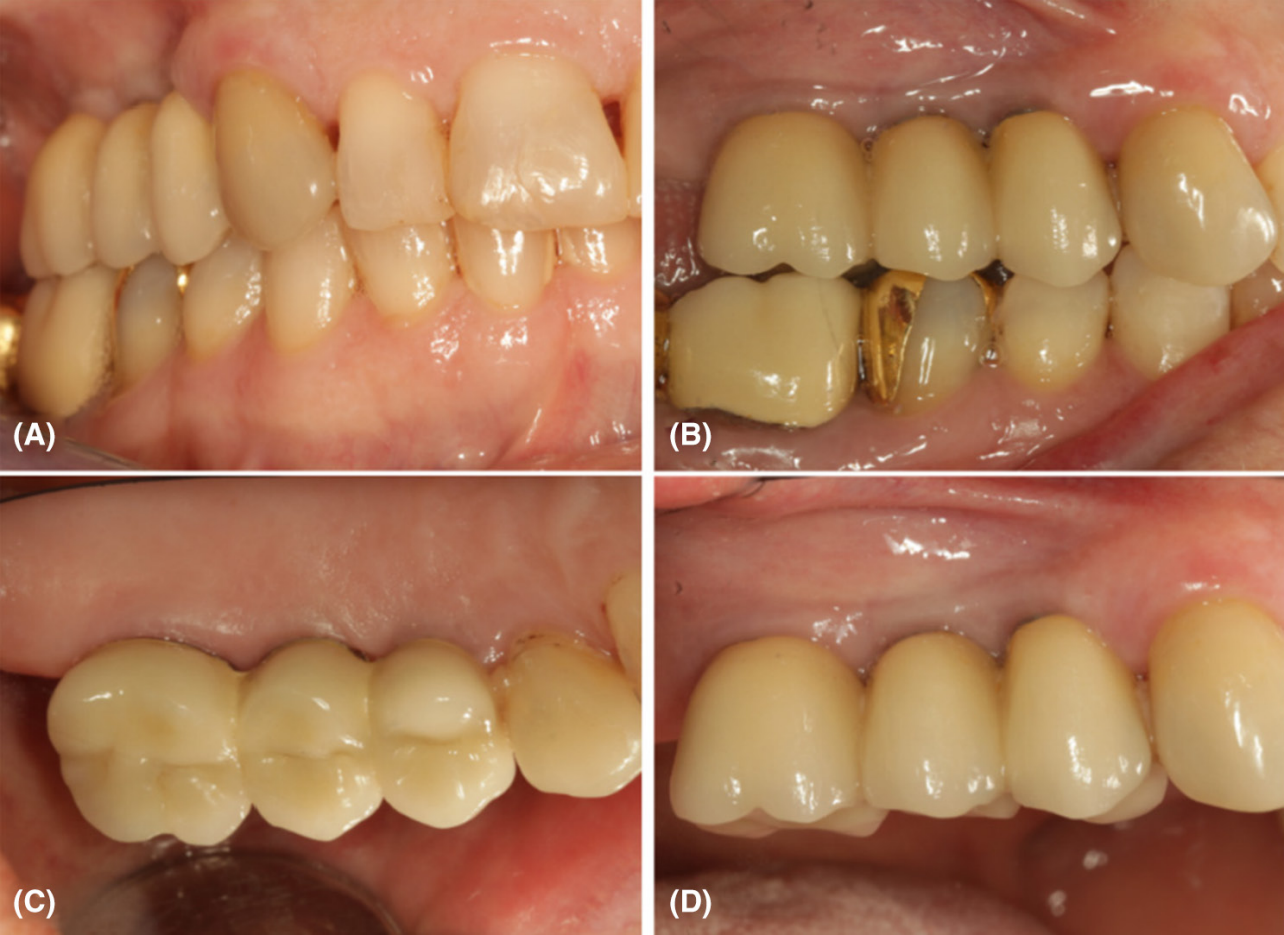
Clinical examination at the 5‐year follow‐up visit: (A) frontal view, (B) occlusion, (C) occlusal view, (D) buccal view.

The annual follow‐up clinical and radiographic examinations showed a stable condition of the examined implants and other parts of the dentition even 5 years after implant insertion (Figures 3 and 4). However, the 2.5‐year clinical examination revealed bleeding on probing at the buccal aspects of the examined implants and increased probing pocket depths around teeth #7, #8, #9, and #10. An additional nonsurgical intervention was needed to overcome this issue, comprised of supra‐ and subgingival plaque removal at teeth #7 –#10 using piezoelectric ultrasonic instruments (PiezoLED ultrasonic scaler with Piezo Scaler tip 203 [KaVo dental]). Due to COVID‐19, one scheduled maintenance session was skipped in 2020 and one in 2021.

In the last year, the patient was diagnosed with two additional medical issues: hypertension and hypothyroidism. As a result, she started taking perindopril 5 mg/day (Bioprexanil, Servier) and levothyroxine 25 mcg/day (Eutirox, Merck). Five years after implant insertion, her bone density (measured with DXA) decreased to a T‐score of −2.1, the diagnosis remaining osteopenia.

## 4 DISCUSSION

Several recent studies have investigated the possibility of rehabilitating shortened maxillary arches with extra‐short implants, finding them to exhibit lower costs, shorter recovery times, minimal invasiveness, and promising med‐term results in cases of single crowns^15^, ^16^, ^17^ as well as splinted crowns in partially edentulous maxillae with gaps between the premolar and distal molar.^18^, ^19^ Similarly, our recent case series yielded excellent 3‐year survival and success rates of splinted crowns supported by one or two extra‐short 4‐mm dental implants and one standard 10‐mm dental implant in shortened maxillary arches, despite the fact that more than half of the treated patients presented with concomitant systemic illnesses, which could potentially compromise implant survival.^13^ The present case report with a 5‐year follow‐up period describes a patient whose bone regeneration capacity was disrupted due to osteopenia and ARD therapy. In addition, the patient refused to undergo any surgical procedure aimed at vertical bone augmentation due to her systemic health and wanted a fixed, minimally invasive option in the shortest possible time.

Despite poor bone quality, extremely low primary implant stability, and the reactivation of herpetic lesions in the early post‐operative period, the stability of the implants improved after 6 months. Although the stability of both 4‐mm implants measured below 60 ISQ–a threshold value that is considered to guarantee safe rehabilitation in splinted restorations,^20^ the prosthetic rehabilitation remained favorable even after 5 years and provided the patient with excellent function. In addition, the 5‐year survival rate of the non‐bicortically stabilized extra‐short implant placed at edentulous site #4 was not compromised even though it has been found that bi‐cortical stabilization is key for short implant stability. Ng et al. showed that only 51% of 6‐mm single‐tooth maxillary implants without bi‐cortical stabilization survived a 5‐year follow‐up period in contrast to 100% of bi‐cortically stabilized 6‐mm single‐tooth implants.^21^

However, short implants should be used cautiously since they are associated with a higher risk of failure compared to standard implants. Only patients with sufficient horizontal bone width, that is, those with a buccal and palatal plate measuring at least 1–1.5 mm, should be considered as candidates for prosthetic rehabilitation with extra‐short implants to avoid critical remodeling of marginal bone.^1^ Splinting is also highly encouraged since it serves as a measure of protection against excessive masticatory forces; in addition, only edentulous regions with no or minor vertical bone resorption seem to be suitable because of the unfavorable crown‐to‐implant ratios at short implant sites.^12^ In the present case study, the crown‐to‐implant ratio was more than 1:2 at the sites of the two extra‐short implants, yet no technical complications were found even at the 5‐year follow‐up visit, most probably because we were very careful when adjusting the occlusal contacts, ensuring disclusion during lateropulsion. Even though the number of case series showing favorable results of 4‐mm implants is on the rise,^15^, ^16^, ^17^, ^18^, ^19^ no RCT has been performed to directly compare the effectiveness and success rates of sinus lifts versus extra‐short implants.

Our patient's medical history revealed that she had been consecutively treated with two ARDs prior to implant placement—first with alendronate, a second‐generation bisphosphonate, for 7 years, followed by denosumab, a human monoclonal antibody, for 2 years. It is known that bisphosphonates remain circulating in the body for up to 10 years even after their discontinuation, resulting in impaired angiogenesis and prolonged inhibition of osteoclast action.^22^ This presented a risk factor for potential bone grafting aimed at augmenting the patient's low maxillary floor since such procedures rely on a functioning vascular recipient site, intact osteoclastic resorption and unimpaired osteoblastic bone formation.^23^ Considering the recommendation that bone augmentations such as sinus lifts should be, if possible, avoided in such cases due to very limited data concerning their safety, success, and risk for osteonecrosis,^6^ prosthetic rehabilitation with extra‐short implants was deemed a better alternative treatment option for our patient. Nevertheless, it has been suggested that dose and cumulative duration of use (>3–4 years) rather than the route of ARD administration is associated with a greater risk for medication‐related osteonecrosis of the jaws (MRONJ).^6^, ^9^, ^23^ Accordingly, Woo et al.^24^ found that approximately 94% of all patients with bisphosphonate‐related osteonecrosis of the jaws received bisphosphonates intravenously (prescribed for the treatment of cancer/metastases) and only 6% orally (prescribed for the treatment of osteoporosis). It is also known that MRONJ is more likely to appear in the mandible.^22^ Therefore, patients with high‐dose ARD intake, patients on oral ARDs over a longer period of time, and patients with comorbidities should be considered as high‐risk candidates for MRONJ.^6^ These patients should be placed on regular long‐term recall schedules.^25^ The American Association Of Oral And Maxillofacial Surgeons, however, still has not reached a consensus regarding the need for a “drug holiday” prior to dentoalveolar surgery in patients receiving oral or intravenous ARD therapy—if, however, the choice were made in favor of treatment suspension, it is recommended that implant insertion be performed 4 months following the last dose of ARD administration.^25^

In cases of osteopenia‐associated poor bone quality, titanium‐zirconium (Ti‐Zr) implants with hydrophilic surfaces may enhance osteointegration compared to pure Ti implants with hydrophobic surfaces.^26^ Ti alloys containing 13%–15% Zr may aid osteointegration in osteoporotic bone, since they have been shown to require a higher removal torque in comparison to pure Ti implants in a rabbit osteoporosis model.^27^ Four‐millimeter implants have narrower thread pitches (0.8 mm) than 10‐mm implants (1.25 mm), which increases their implant‐to‐bone contact ratios.

## 5 CONCLUSION

After 5 years of strict maintenance, two 4‐mm extra‐short tissue level Ti‐Zr implants, splinted to a standard‐size implant, enabled the successful rehabilitation of our patient's shortened maxillary dental arch despite poor bone quality, low primary implant stability, osteopenia, and a history of ARD therapy. Within the limitation of the study, extra‐short 4‐mm implants splinted to a standard‐size implant may be used as a less invasive, faster, and more economical alternative compared to sinus lifts. However, additional trials with a high number of participants and extended observation periods are needed to provide guidelines for such treatment concepts.

## AUTHOR CONTRIBUTIONS

**Katja Povšič:** Data curation; project administration; writing – original draft; writing – review and editing. **Čedomir Oblak:** Data curation; investigation; methodology; writing – original draft; writing – review and editing. **Michel Dard:** Project administration; writing – original draft; writing – review and editing. **Rok Gašperšič:** Conceptualization; data curation; formal analysis; investigation; methodology; project administration; supervision; writing – original draft; writing – review and editing.

## CONFLICT OF INTEREST STATEMENT

No conflict of interest relevant to this article is reported by the authors.

## CONSENT

Written informed consent was obtained from the patient to publish this report in accordance with the journal's patient consent policy.

## Data Availability

Data sharing not applicable to this article as no datasets were generated or analysed during the current study.
